# The Role of CO_2_ Levels in High-Oxygen Modified Atmosphere Packaging on Microbial Communities of Chilled Goat Meat During Storage and Their Relationship with Quality Attributes

**DOI:** 10.3390/foods14111837

**Published:** 2025-05-22

**Authors:** Samart Sai-Ut, Sylvia Indriani, Nattanan Srisakultiew, Passakorn Kingwascharapong, Sarisa Suriyarak, Utthapon Issara, Suphat Phongthai, Saroat Rawdkuen, Jaksuma Pongsetkul

**Affiliations:** 1 Department of Food Science, Faculty of Science, Burapha University, Chonburi 20131, Thailand; samarts@go.buu.ac.th; 2 School of Animal Technology and Innovation, Institute of Agricultural Technology, Suranaree University of Technology, Nakhon Ratchasima 30000, Thailand; indrianisylvia@gmail.com (S.I.); nattanan.s@sut.ac.th (N.S.); 3 Department of Fishery Products, Faculty of Fisheries, Kasetsart University, Bangkok 10900, Thailand; passakorn.ki@ku.ac.th; 4 Department of Food Technology, Faculty of Food Science, Chulalongkorn University, Bangkok 10330, Thailand; sarisa.s@chula.ac.th; 5 Division of Food Science and Technology Management, Faculty of Science and Technology, Rajamangala University of Technology Thanyaburi, Pathum Thani 12110, Thailand; utthapon_i@rmutt.ac.th; 6 Faculty of Agro-Industry, Chiang Mai University, Chiang Mai 50200, Thailand; suphat.phongthai@cmu.ac.th; 7 Food Science and Technology Program, School of Agro-Industry, Mae Fah Luang University, Chiang Rai 57100, Thailand; saroat@mfu.ac.th

**Keywords:** goat meat, high-oxygen modified atmosphere packaging, microbial communities, quality attributes

## Abstract

This study investigated the influence of CO_2_ levels (20–40%: M20, M30, and M40) in high-oxygen modified atmosphere packaging (Hi-O_2_ MAP) on microbial communities and quality attributes of chilled goat meat stored at 4 °C for 12 days. Alpha diversity indices (Chao1, ACE, Simpson, and Shannon) revealed a significant decline in microbial diversity over time, with storage duration exerting a greater impact than packaging conditions. Nonetheless, MAP played a crucial role in shaping microbial profiles, with air packaging (AP) showing the most distinct community, while M40 differed notably from M20 and M30, particularly by day 12, as shown by beta diversity analysis using principal coordinates analysis (PCoA). *Proteobacteria* and *Firmicutes* dominated microbial composition, with *Pseudomonas* and *Brochothrix* linked to spoilage in AP, while MAP, especially M40, favored the growth of *Lactococcus*, *Acinetobacter*, and *Vagococcus*, enhancing microbial stability. Despite pathogen levels remaining within safe limits, AP exceeded the spoilage threshold (TVC > 7.00 log colony-forming unit (CFU)/g), whereas all MAPs extended shelf life, with M40 most effectively suppressing microbial growth (*p* < 0.05). Interestingly, metagenomic functional profiling revealed that elevated CO_2_ levels (>30%) altered metabolic pathways, shifting spoilage mechanisms from protein degradation in AP to carbohydrate metabolism in MAP, potentially influencing odor and texture attributes. MAP, particularly M40, also reduced protein and lipid degradation and oxidation, as indicated by lower total volatile base nitrogen (TVB-N), thiobarbituric acid reactive substances (TBARSs), and shear force, suggesting better prevention of increased meat hardness and the development of undesirable odors and flavors, although high CO_2_ negatively affected redness. Overall, M40 provided the greatest microbial stability and shelf life extension, highlighting the potential of optimized CO_2_ levels in Hi-O_2_ MAP to preserve goat meat quality and regulate spoilage dynamics.

## 1. Introduction

Goat meat, valued for its leanness and distinct flavor, is widely consumed in Asia, Africa, and the Middle East [1]. Goat farming supports sustainable food systems, as goats efficiently convert low-quality feed and thrive in harsh environments, making them essential in rural areas [1,2]. Rising global demand reflects its nutritional benefits, with the goat population reaching 1.128 billion in 2020 [3]. However, goat meat is highly perishable, lasting only 6–12 days under chilled storage due to microbial and biochemical spoilage [4,5,6]. This short shelf life is largely due to microbial growth and biochemical deterioration, underscoring the need for effective preservation strategies to maintain quality and safety. Modified atmosphere packaging (MAP) is widely used to extend fresh meat shelf life by adjusting gas composition: O_2_ maintains color, CO_2_ inhibits microbial growth, and N_2_ prevents oxidation and package collapse [7]. High-oxygen MAP (Hi-O_2_ MAP), with 50–80% O_2_, is common for red meats to stabilize oxymyoglobin, giving the meat its bright red color [8], with 50% O_2_ considered optimal for balancing color retention and oxidation [9]. While O_2_’s role is well understood, optimal CO_2_ levels are less defined; 20–40% CO_2_ is generally effective for microbial control without risking package collapse, which may occur above 40% [10]. CO_2_ also modulates microbial community dynamics, influencing spoilage pathways and meat quality.

The gas composition in MAP plays a critical role in shaping bacterial succession, modifying microbial community structure and the characteristics of spoilage over time. Changes in packaging conditions can lead to distinct microbial shifts and spoilage outcomes. Under aerobic storage, *Pseudomonas* dominates and drives spoilage, whereas CO_2_-enriched or anaerobic conditions favor the growth of Gram-positive bacteria such as lactic acid bacteria (LAB) and *B. thermosphacta* [11]. Interestingly, despite being aerobic and CO_2_-sensitive, *Pseudomonas* has been shown to adapt to MAP environments, reaching high counts in minced beef and contributing to spoilage [12]. Spoilage microbiota developed in a somewhat unpredictable manner during storage, influenced by the differing metabolic activities of individual species [6]. However, the specific roles of these microbes remain unclear, highlighting the need for further investigation. High-throughput DNA sequencing has emerged as a powerful tool for analyzing microbial community dynamics, offering higher resolution than conventional culture-based methods [13]. While numerous studies have explored the impact of MAP on the microbiota of beef, pork, poultry and seafood [14,15,16,17], comparable studies on goat meat remains limited. Although Carrizosa et al. [11] identified *Enterobacteriaceae* as dominant in MAP-stored goat meat, their study did not explore long-term microbial succession or the relationship between microbial shifts and biochemical or physicochemical changes. Furthermore, the role of CO_2_ levels in shaping microbial communities under Hi-O_2_ MAP conditions and their subsequent effects on goat meat quality has not been extensively investigated. To address this gap, the present study utilized high-throughput DNA sequencing and metagenomic analysis to examine microbial metabolic pathways in goat meat stored in Hi-O_2_ MAP with varying CO_2_ concentrations (20%, 30%, and 40%) at 4 °C over a 12-day period. Additionally, principal component analysis (PCA) was performed to assess correlations between microbial profiles and key quality attributes, including color stability, lipid oxidation, pH, and texture. These integrated approaches aim to elucidate how CO_2_ levels influence microbial succession and spoilage mechanisms, ultimately helping to identify the optimal gas composition for maximizing the shelf life and quality of goat meat under Hi-O_2_ MAP conditions.

## 2. Materials and Methods

### 2.1. Chemicals

Peptone water (0.1%) and plate count agar (PCA) were obtained from Merck (Darm-stadt, Germany). Reagents for total volatile base nitrogen (TVB-N) analysis, including boric acid (H_3_BO_3_) and hydrochloric acid (HCl), were of analytical grade and purchased from Sigma-Aldrich (St. Louis, MO, USA). For lipid oxidation analysis, thiobarbituric acid (TBA) and malonaldehyde bis(dimethyl acetal) standards were also procured from Sigma-Aldrich. All other chemicals, including distilled water, were of analytical grade and used as required in the respective analytical procedures.

### 2.2. Sample Preparation and Packaging Conditions

*Longissimus thoracis* (LT) loin muscles were obtained from 39 male crossbred goats (*Thai-native* × *Anglo-Nubian*), aged between 6 and 8 months, with carcass weights ranging from 9.95 to 12.41 kg. The animals were sourced from multiple commercial farms in Nakhon Ratchasima province and processed at a commercial slaughterhouse. After slaughter, the loins were immediately placed in an ice box maintained at −2 to 1 °C and transported to the laboratory within 1 h. Upon arrival, they were stored at 4 °C for 48 h. Surface fat and connective tissues were then trimmed off, and the loins were sliced into 1.5 cm-thick pieces, each weighing around 150–180 g, for subsequent packaging treatments. The experiment was conducted in three separate batches, each containing 13 goat loin samples. From each batch, 65 slices were prepared. Five slices were retained as Day 0 samples for analysis, while the remaining 60 slices were divided into 4 treatment groups (15 pieces/treatment) and packed under different MAP conditions. We performed MAP condition using a Henkovac type 1000 (Tecnovac, Grassobbio, Italy) with a gas mixture including M20: 20% CO_2_/50% O_2_/30% N_2_, M30: 30% CO_2_/50% O_2_/20% N_2_, and M40: 40% CO_2_/50% O_2_/10%, with an air-packed group serving as the control (AP). For packing, each meat slice was individually placed on a polyethylene terephthalate (PET) tray (18 × 13 × 4 cm^3^ with a thickness of 0.25 mm), with O_2_, N_2_, and CO_2_ permeability values of 2.5, 2.5, and 45 cm^3^/m^2^·day·atm, respectively, and a water vapor permeability of <1 g/m^2^·day (85% RH, 25 °C). The desired gas mixtures were introduced into the trays, maintaining a gas-to-meat volume ratio of 3:1. The trays were then sealed using a multilayer polyamide (PA)/ethylene vinyl alcohol (EVOH) MAP film (O_2_ transmission rate <1 cm^3^/m^2^·day·atm; Greenpak, Jiangyin, China) using a Henkovac tray sealer (Type 1000, Tecnovac, Grassobbio, Italy). During storage at 4 °C for 12 days, samples were collected for analysis on Days 0 (D0), 6 (D6), and 12 (D12).

### 2.3. Determination of Microbial Community (Microbiome Analysis)

Genomic DNA was isolated from three samples per treatment group using the FavorPrep Stool DNA Isolation Mini Kit (Favorgen, Ping-Tung, Taiwan), following the manufacturer’s instructions. DNA quality was evaluated via 1% agarose gel electrophoresis, and concentrations were determined using a NanoDrop 2000 spectrophotometer (NanoDrop Technologies, Wilmington, DE, USA). Library preparation, sequencing, and bioinformatics analyses were carried out by Novogene (Beijing, China). In total, seven DNA libraries were constructed to amplify the V3–V4 regions of the 16S rRNA gene. Following library preparation, DNA quantification was performed using the Qubit dsDNA HS Reagent Kit, and sequencing was conducted on the Illumina HiSeq 2500 platform (Illumina Inc., San Diego, CA, USA) with 2 × 250 bp paired end reads, adhering to standard protocols. Raw sequencing reads were quality-filtered and processed using Mothur software (Version 1.48.0, University of Michigan, Ann Arbor, MI, USA) [18] to remove low-quality reads and chimeras. Operational taxonomic units (OTUs) were classified based on the Greengenes database (Version 13_8) at 97% similarity. Rarefaction curves were generated to evaluate sequencing depth and species richness in each sample. The alpha diversity (Chao1, Simpson, and Shannon indices) were estimated and tested using the Mann–Whitney test (nonparametric *t*-test) with a 95% level of confidence. Principal Coordinates Analysis (PCoA), Venn diagrams, and hierarchically clustered dendrogram were conducted to examine the similarities or differences of the microbiome across various treatments. Graphical representation of the relative abundance of microbial diversity was visualized using the ampvis2 R-package in RStudio (Version 2023.12.0, Boston, MA, USA). Functional gene prediction of microbial communities was carried out using the retained OTUs from the predicted metagenomes through the PICRUSt tool (Version 1.1.4) [19]. The predicted genes were then assigned to biological functions based on the Kyoto Encyclopedia of Genes and Genomes (KEGG) pathway database (Kyoto, Japan) (http://www.kegg.jp/kegg/pathway.html, accessed on 15 January 2025).

### 2.4. Determination of Total Viable Count (TVC) and Pathogen

For TVC, a 25 g of each sample was mixed with 225 mL of 0.1% peptone water and homogenized at high speed for 3 min using a Stomacher 400 Lab Blender (Seward Ltd., Worthing, UK). Serial tenfold dilutions were then prepared, and the appropriate dilutions were plated on Plate Count Agar (Merck, Rahway, NJ, USA). The plates were incubated at 37 °C for 3 days, and results were expressed as colony-forming unit (CFU)/g sample [20]. For pathogens, *Escherichia coli*, *Staphylococcus aureus*, *Clostridium perfringens*, *Salmonella* spp., and *Campylobacter jejuni*, were counted following the Thai agricultural commodity and food standard (ACFS, Bangkok, Thailand) [21].

### 2.5. Determination of Quality Attributes

The pH was measured by blending 10 g of ground sample with 100 mL of distilled water, using a pH meter (Sartorius, Model PB-10, Göttingen, Germany). Redness (a*) was measured at seven different spots on the sample using a chromameter (Konica Minolta, Model CR-410, Tokyo, Japan). Total volatile base nitrogen (TVB-N) content was measured using the Conway microtitration method and reported as mg N/g sample [22]. Lipid oxidation was determined using the thiobarbituric acid reactive substances (TBARSs) method described by Pongsetkul et al. [23]. A standard curve was prepared using malonaldehyde bis(dimethyl acetal) at concentrations ranging from 0 to 2 ppm. TBARS values were expressed as mg of malonaldehyde (MDA)/kg sample. Shear force was assessed using a Texture Analyzer (Stable Micro Systems, Model TA.XT Plus, Surrey, UK). Samples were cut into cubes measuring 1 × 2 × 1 cm^3^, with muscle fibers aligned along the longitudinal axis. A Warner–Bratzler blade was used for the test, operated at a speed of 4 mm/s with a 50 kg load cell [24].

### 2.6. Statistical Analysis

A completely randomized design (CRD) was applied, with three independent batches allocated to each treatment group. Each batch was processed and analyzed separately to ensure true replication. Results are presented as mean ± standard deviation. Statistical analysis was conducted using two-way analysis of variance (ANOVA), followed by Tukey’s post-hoc test to identify significant differences among treatment means at a 95% confidence level, using SPSS software (Version 25.0, IBM Corp., Chicago, IL, USA). Additionally, principal component analysis (PCA) was performed using XLSTAT software (Version 2018.7, Addinsoft, New York, NY, USA) to explore the associations between dominant microbial communities and key quality parameters of samples stored under different MAP conditions.

## 3. Results and Discussion

### 3.1. Dynamic Changes in Microbial Communities During Storage

#### 3.1.1. Microbial Richness and Diversity in Goat Meat Stored Under Map Conditions

To gain a clear understanding of changes microbial diversity and abundance under MAP conditions, high-throughput sequencing of bacterial communities in chilled goat meat was performed using the Illumina MiSeq platform. A total of 488,780 high-quality reads were obtained across seven libraries, with read counted from 49,299 to 57,340 reads (Table 1). These sequences were clustered into 3737 OTUs and classified into 47 phyla, 101 classes, 299 orders, 472 families, 889 genera, and 1253 species based on the Greengenes database at a 97% similarity threshold (Table S1). Sequencing depth exceeded 97% coverage for all samples, indicating comprehensive detection of bacterial populations (Table 1).

Alpha diversity indices, including Chao1, ACE, Simpson, and Shannon, were used to assess microbial richness and evenness within individual samples. At Day 0, fresh goat meat exhibited the highest microbial richness and diversity, with the highest OTU count (492), Chao1 (922), Simpson (0.96), and Shannon (6.02) values, suggesting a more diverse microbial population compared to samples during storage. Over time, microbial richness and diversity declined at varying rates among the samples. Interestingly, the AP sample showed a sharper drop in OTUs, decreasing to 366 by Day 12, yet it retained higher Chao1, ACE, and Simpson values compared to MAP samples. This rapid drop in OTUs, coupled with sustained high diversity indices, suggested that AP initially harbored many rare taxa, which were lost over time, while a stable and even microbial community persisted during storage under atmospheric conditions. Under MAP conditions, microbial diversity was generally suppressed. M20 and M30 showed lower richness than M40 on both Day 6 and Day 12, as indicated by lower alpha diversity indices. This suggested that 20–30% CO_2_ selectively inhibited bacterial growth, whereas 40% CO_2_ might have supported the development of distinct microbial communities. Moreover, the results showed that M40 exhibited higher or comparable microbial evenness to AP, as reflected by similar or higher Shannon and Simpson indices, both of which were greater than those of M20 and M30. This indicated that M40 supported a more balanced microbial community, which may help slow spoilage. Although storage time was the primary factor influencing microbial changes, packaging conditions, especially higher CO_2_ levels in M40, also played a significant role. These conditions likely shaped microbial dynamics by regulating bacterial succession and spoilage pathways, thereby potentially extending shelf life [14,25]. Similar to our findings, Chen et al. [26] reported that *Firmicutes* and *Bacteroidetes* dominated in fermented goat sausage, accounting for over 66% of all OTUs, with microbial diversity decreasing over time. A similar trend of declining microbial diversity has also been observed in various meat products such as portly, oysters, bacon and beef [13,14,16,17], where specific packaging conditions favored certain bacterial groups, leading to shifts in microbial communities.

**Figure 1:**
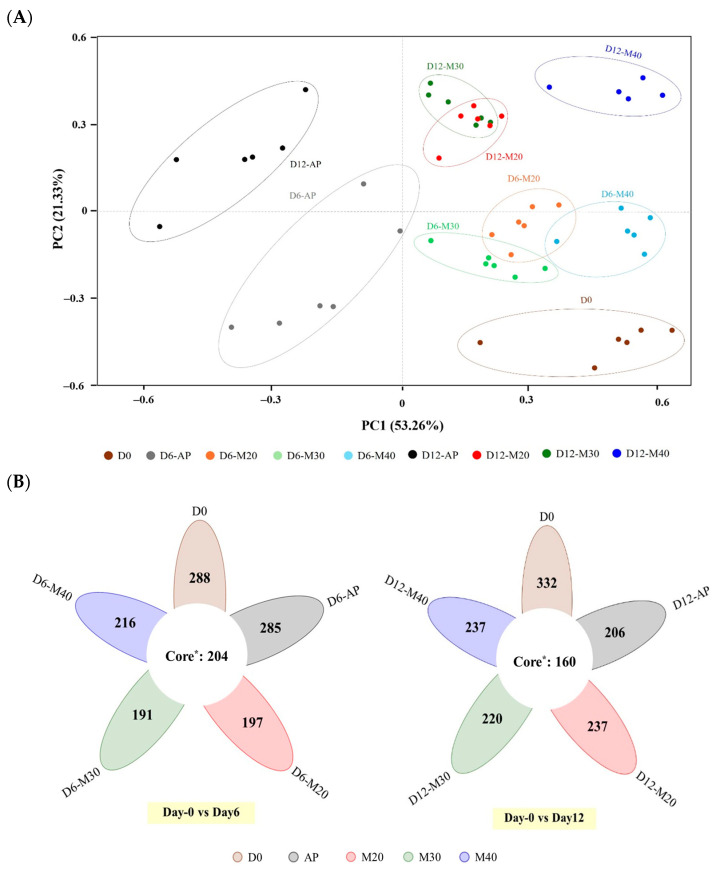
Beta diversity (PCoA plot) (**A**), and Venn diagram (**B**) of bacterial communities in goat meat stored under different MAP conditions at 4 °C for 12 days. * Core: OTUs commonly found in all samples.

Beta diversity, which refers to the difference in species composition between samples, was assessed using PCoA, revealing distinct clustering patterns that explained 74.59% of the total variation (Figure 1A). PC1 (53.26%) showed a clear separation between AP samples from Day 6 and Day 12 (left side) and all MAP samples (right side), emphasizing the dominant effect of packaging on microbial composition. This suggested that packaging plays a critical role in shaping microbial communities, which in turn impacts meat quality and safety. Microbial composition of AP deviated the most from Day 0, as early as Day 6, suggesting that rapid spoilage and quality changes occurred in meat stored under atmospheric conditions, while all MAP treatments were more effective at retarding these changes. These findings align with previous reports demonstrating that MAP can slow microbial succession compared to aerobic conditions [25,27,28]. PC2 (21.33%) reflected the influence of storage time, as Day 12 samples positioned in the upper region, while earlier samples remained in the lower region. Interestingly, M20 and M30 samples exhibited close clustering with minimal dispersion, suggesting similar microbial communities and reduced diversity over time. In contrast, M40 samples showed greater variation, indicating a more dynamic shift in microbial communities during storage, which may correspond to their distinct quality attributes under 40% CO_2_ conditions. The Venn diagram (Figure 1B) further supported these observations, illustrating shared and unique OTUs among different storage days. On Day 6, 204 OTUs were common across all samples, but this number declined to 160 by Day 12, suggesting that microbial shifts depend on packaging conditions due to extended storage. Remarkably, AP samples exhibited the highest number of unique OTUs on Day 6, but by Day 12, they had the lowest, reinforcing the idea that microbial communities in MAP samples remained more stable over time.

#### 3.1.2. Microbial Community Composition and Abundance

**Figure 2:**
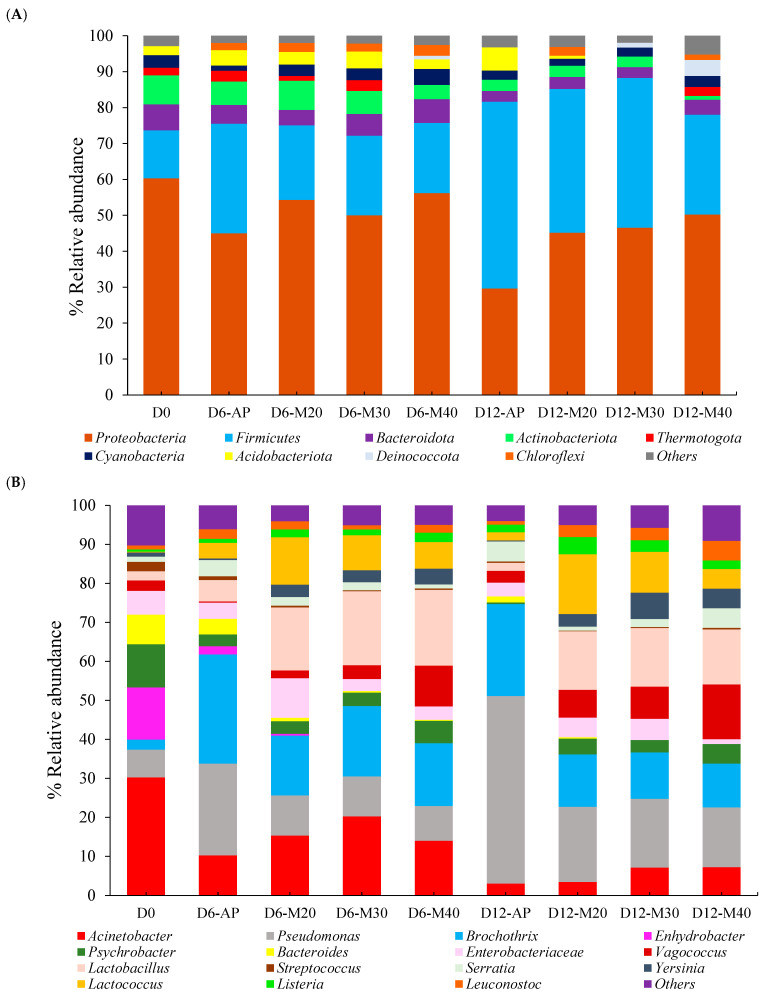
Relative abundance (%) at phylum (**A**) and genus (**B**) levels of bacterial communities in goat meat stored under different MAP conditions at 4 °C for 12 days.

At the phylum level (Figure 2A), *Proteobacteria* was the dominant phylum in all samples (>45%), except for AP on Day 12, where its abundance decreased to 29.63%, while *Firmicutes* became predominant (51.99%). Together, these two phyla accounted for over 70% of all bacterial communities, with their relative abundances influenced by packaging conditions and storage duration. This pattern was consistent with previous studies that identified them as the primary microbial groups in both fresh and spoiled meat [13,14,17]. A notable shift between these two phyla was observed over time, with *Proteobacteria* decreasing and *Firmicutes* increasing, particularly in AP samples. This trend suggested a microbial succession process, where spoilage conditions favor *Firmicutes*, while the decline in *Proteobacteria* may indicate a reduction in spoilage-related bacteria commonly associated with aerobic conditions. In this study, AP contained lower oxygen levels than MAPs (50% O_2_), which likely accelerated the growth of *Firmicutes*, as this phylum generally thrives under anaerobic conditions. It includes many anaerobic or facultatively anaerobic bacteria, such as *Clostridium* and *Lactobacillus*, that are well adapted to low-oxygen environments, especially during meat storage and spoilage [29]. In MAP samples, the proportion of Firmicutes also increased over time, though at a slower rate, particularly in M40. Yang et al. [14] reported a significant increase in *Firmicutes* under both high-O_2_ MAP (80% O_2_/20% CO_2_) and high-CO_2_ MAP (0.4% CO/30% CO_2_/69.6% N_2_), indicating beef steak spoilage. This increase was attributed to the outgrowth of the dominant genera *Brochothrix thermosphacta* and *Lactococcus* under each condition, respectively. These findings support our results, suggesting that the lower increase of *Firmicutes* observed in M40 may reflect a slower spoilage rate in goat meat under this condition. Other phyla, such as *Bacteroidota, Actinobacteria*, and *Thermotogota*, were initially detected in fresh goat meat (Day 0) but declined or disappeared in certain packaging treatments over time. In contrast, *Deinococcota* increased in M40, suggesting a potential adaptation to high-CO_2_ conditions. By Day 12, M40 had the lowest abundance of *Acidobacteriota* compared to the other treatments. Notably, M40 also exhibited the highest proportion of other bacterial phyla (5.23%), indicating a more diverse and distinct microbial community. This may reflect the unique influence of 40% CO_2_ in shaping the microbial profile, potentially affecting meat quality during storage, distinct from the effects observed with 20–30% CO_2_ or atmospheric conditions.

At the genus level (Figure 2B), microbial diversity was highest in fresh goat meat (Day 0) and declined with storage. The microbial profiles of AP samples were distinct from MAP samples, reinforcing the influence of packaging over time. *Acinetobacter* was the most dominant genus in fresh meat (30.22%) but declined as storage progressed, regardless of packaging conditions (3.07–7.22% on Day 12). This aerobic bacterium is often associated with fresh meat but is less competitive under storage conditions that favor anaerobic or facultative anaerobic bacteria [30]. In contrast, *Pseudomonas* and *Brochothrix* were initially present at lower levels (7.16% and 2.55%, respectively) but increased over time, particularly in AP. By Day 12, *Pseudomonas* accounted for 48.02% of the AP microbial community, highlighting its role as a dominant spoilage bacterium under aerobic conditions. This aligned with previous studies showing that *Pseudomonas* is a key contributor to spoilage in meat stored under air due to its proteolytic and lipolytic activity, which accelerates quality deterioration [10,31,32]. The lower abundance of *Pseudomonas* in MAP suggested that CO_2_ can effectively suppress its growth, thus extending shelf life. Interestingly, *Brochothrix* became highly abundant in AP from Day 6 onward, whereas MAP selectively promoted the growth of *Lactobacillus* and *Lactococcus,* which remained above 10% and 5%, respectively, throughout storage. The consistent presence of *Lactobacillus* in MAP samples suggested a beneficial role in meat preservation, as it can outcompete spoilage bacteria and produce antimicrobial metabolites that help maintain meat quality, particularly in terms of flavor and odor. These LAB are commonly found in MAP meat products and contribute to quality preservation by producing organic acids that inhibit spoilage microorganisms [33]. Their presence is often associated with improved odor and flavor stability due to reduced proteolysis and lipid oxidation [34]. Furthermore, the suppression of *B. thermosphacta*, a bacterium commonly linked to off-odors in meat, highlighted the superior ability of MAPs, particularly with higher CO_2_ concentrations, to preserve flavor and limit spoilage. Notably, by Day 12, the microbial composition of M40 was distinctly different from that of M20 and M30. M40 exhibited lower levels of Lactococcus but higher abundances of *Vagococcus* (14.06%), *Psychrobacter* (5.02%), and *Leuconostoc* (4.99%). While *Leuconostoc* is often linked to LAB-driven fermentation and improved sensory attributes, *Psychrobacter* has been linked to off-odors and spoilage under cold storage [16]. The presence of *Vagococcus* in M40 suggested a shift in microbial dynamics that may influence both beneficial and undesirable quality traits [35]. These results confirmed a unique microbial pattern associated with the increase in CO_2_ to 40% in high-O_2_ MAP, which may contribute to distinct changes in meat quality during storage. Furthermore, it also highlighted the importance of CO_2_ levels in regulating the microbial pattern to preserve meat quality.

**Figure 3:**
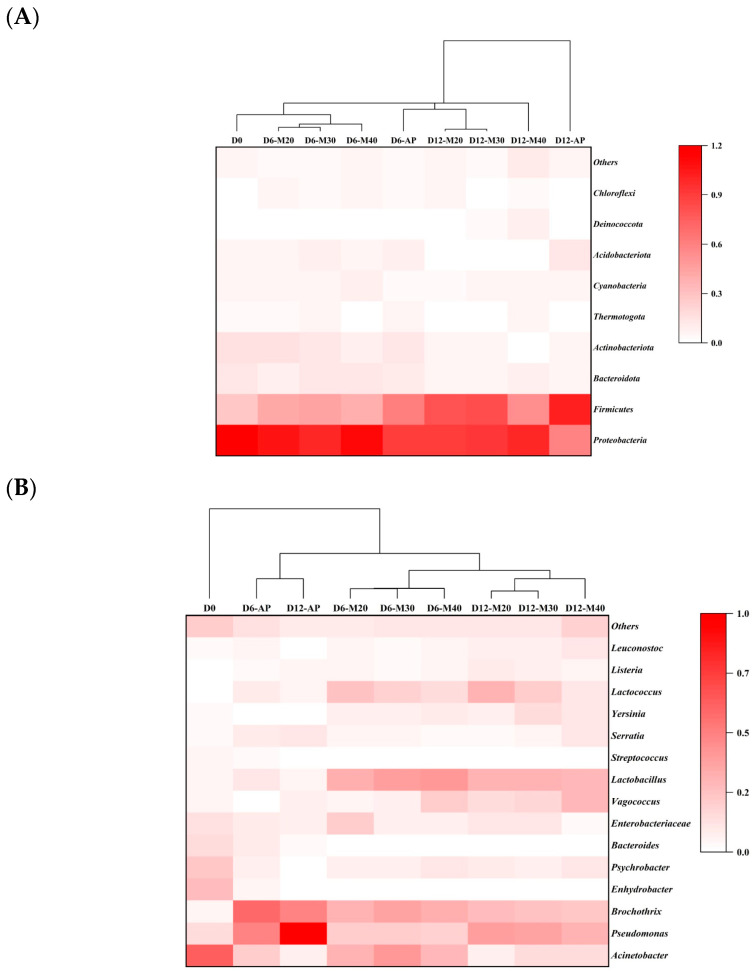
Hierarchically clustered dendrogram of bacterial communities at phylum (**A**) and genus (**B**) levels of bacterial communities in goat meat stored under different MAP conditions at 4 °C for 12 days.

The hierarchical clustering dendrograms at both the phylum and genus levels are shown in Figure 3A and Figure 3B, respectively. The correlation dendrogram confirmed distinct microbial distributions that were strongly influenced by packaging. Notably, D0 was clearly separated from the others, while within the remaining samples, all MAP samples clustered together, and AP samples exhibited unique microbial patterns. In particular, two phyla including *Proteobacteria* and *Firmicutes*, along with ten genera, including *Acinetobacter*, *Pseudomonas*, *Brochothrix*, *Enterobacteriaceae*, *Vagococcus*, *Lactobacillus*, *Serratia*, *Yersinia*, *Lactococcus*, and others, showed high abundance (intensity > 0.3 or abundance > 5%) in at least one sample on Day 12 of storage, suggesting their potential for differentiating the microbial communities among samples. These twelve microbial taxa were therefore selected for correlation analysis with quality attributes.

#### 3.1.3. Metagenomic Functional Profiling

**Figure 4:**
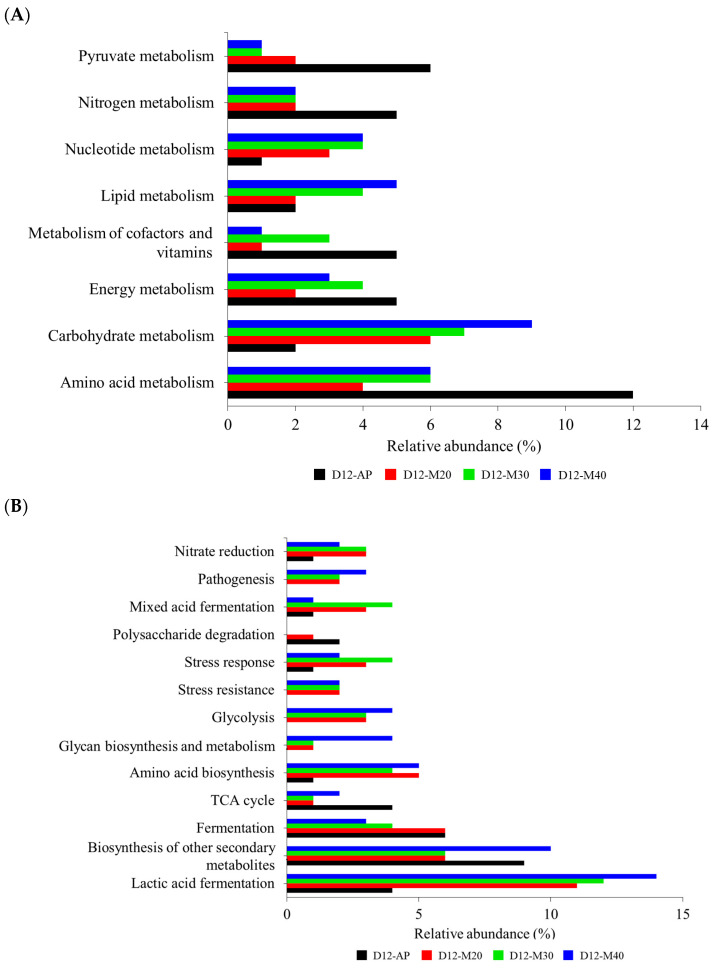
Functional genes related to microbial metabolism (**A**) and other microbial biological functions (**B**) in goat meat stored under different MAP conditions at 4 °C on day 12th of storage.

To gain deeper insights into microbial patterns under different packaging treatments, particularly their impact on molecular pathways involved quality changes, functional gene analysis was performed using microbial profiles from Day 12. The shelf life of meat is influenced not only by the total bacterial load but also by microbial metabolic activity, as spoilage is primarily driven by the accumulation of degradation compounds [36]. Thus, understanding bacterial metabolic pathways through predicted 16S rRNA sequencing functions is essential. Figure 4A and Figure 4B revealed functional genes related to microbial metabolism and other biological functions, respectively. The AP and MAP samples exhibited distinct metabolic profiles, with AP showing dominant pathways in amino acid metabolism (12%), secondary metabolite biosynthesis (9%), and pyruvate metabolism (6%), consistent with spoilage mechanisms under normal air conditions. In AP packaging, O_2_ exposure combined with low CO_2_ levels promotes the growth of aerobic spoilage bacteria such as *Pseudomonas*, *Acinetobacter*, and *Shewanella*, which rely on proteolysis and oxidative metabolism for survival [37]. These metabolic pathways are closely linked to proteolytic activity, with *Pseudomonas*, the predominant genus in AP, utilizing amino acid degradation for energy and producing nitrogenous spoilage compounds such as ammonia and biogenic amines [38]. The high availability of proteins and amino acids in meat supports these metabolic activities by serving as an optimal nutrient source [39]. These findings suggested that AP facilitates spoilage primarily through protein and amino acid degradation. Conversely, MAP samples exhibited dominant pathways in lactic acid fermentation (11–14%) and carbohydrate metabolism (6–9%), likely due to increased prevalence of LAB such as *Lactobacillus*, which ferment carbohydrates into organic acids. This metabolic shift can lower pH, suppress spoilage bacteria, and influence meat texture and flavor [40]. As is typical in MAP systems, the presence of CO_2_ in all high-O_2_ MAP treatments used in this study suppressed anaerobic competitors, allowing O_2_-tolerant species to dominate the microbial community and accelerate spoilage in the manner distinct from that observed under normal air packaging. This was reflected in the detection of pathways related to lipid metabolism, nitrate reduction, pathogenesis, stress response and resistance, glycolysis, glycan biosynthesis and metabolism, as well as amino acid biosynthesis, which were found at higher levels in MAP samples and were either absent or barely detectable in AP samples. Notably, among MAP treatments, M40 favored carbohydrate metabolism and glycolysis, likely due to CO_2_-induced stress altering microbial metabolism. In contrast, M20 and M30 exhibited more dominant pathways in mixed acid fermentation and stress response, suggesting microbial adaptation to CO_2_ enrichment. The results suggested that AP promoted spoilage through proteolytic activity and amino acid degradation, while MAP (particularly with higher CO_2_ levels) mitigated these effects by favoring carbohydrate metabolism and stress-related pathways, depending on CO_2_ levels. Not only did MAP extend shelf life by inhibiting total microbial growth, but its metabolic byproducts could also impact meat quality, warranting further investigation into sensory and physicochemical implications.

### 3.2. Changes in TVC and Pathogen of Goat Meat During Storage

**Figure 5:**
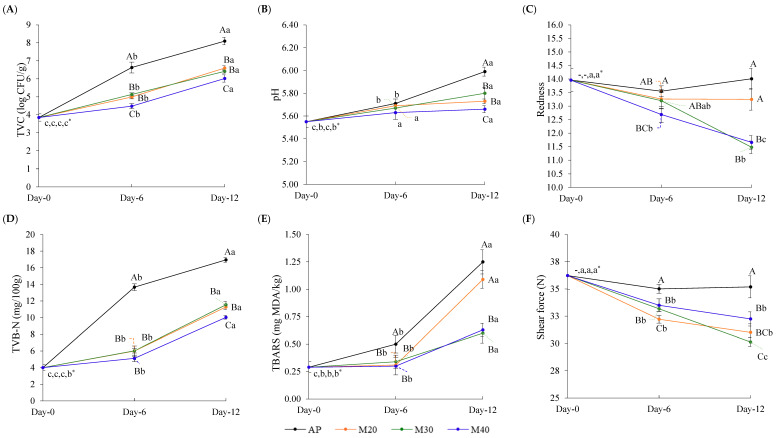
Changes in TVC (**A**), pH (**B**), redness (**C**), TVB-N (**D**), TBARSs (**E**), and shear force (**F**) in goat meat stored under different MAP conditions at 4 °C for 12 days. Different uppercase letters denote significant differences due to MAP conditions at the same storage time (*p* < 0.05). Different lowercase letters denote significant differences across storage durations (*p* < 0.05). * The orders of lowercase letters are for AP, M20, M30, and M40, respectively.

TVC of all samples increased from 3.85 log CFU/g at Day 0, with varying rates as storage time progressed (*p* < 0.05) (Figure 5A). AP samples exhibited higher TVC than MAP samples at both Day 6 and Day 12 (*p* < 0.05), exceeding the spoilage threshold (>7.00 log CFU/g, [41]) within 12 days. This indicated faster deterioration and a shelf life of less than 12 days under normal air condition. As storage progressed, bacteria transitioned from the lag to the exponential growth phase, utilizing proteins, amino acids, and carbohydrates [42]. By day 12, dominant spoilage bacteria detected in AP including *Pseudomonas*, *Brochothrix*, and *Shewanella*, likely played a significant role in accelerating degradation and driving quality changes, with their accumulation exceeding safety limits of fresh meat products. All MAP conditions effectively delayed spoilage, as they resulted in lower TVC compared to AP throughout storage, with M40 demonstrating the most pronounced inhibitory effect (*p* < 0.05). The TVC of 7.00 log CFU/g at Day 12 indicated that MAP products were within standard regulations. The increase in TVC of MAP samples can be attributed to the fact that CO_2_ initially suppressed bacterial growth, but CO_2_-tolerant bacteria (e.g., *Enterobacteriaceae*, LAB) gradually adapted and proliferated. Meanwhile, endogenous proteases broke down muscle proteins, providing nutrients that fueled bacterial expansion [43]. The accumulation of metabolic byproducts (e.g., ammonia, biogenic amines) altered pH, further promoting microbial growth. High CO_2_ concentrations, particularly 40%, reduced TVC more effectively during storage, likely due to CO_2_ creating acidic conditions that disrupt bacterial metabolism and reduce protein degradation [27]. These shifts in microbial patterns and metabolic activities helped delay spoilage. Indriani et al. [17] described that limiting O_2_ in MAP suppressed aerobic bacteria like *Pseudomonas* and *Brochothrix*, while promoting the growth of CO_2_-tolerant bacteria such as *Lactobacillus*, which are less spoilage prone. This suggested that increasing CO_2_ concentrations plays a critical role in microbial suppression, likely through its bacteriostatic properties that reduce bacterial proliferation and metabolic activity.

Pathogen counts are reported in Table 2. At Day 0, the detection of all pathogens in goat meat was either undetectable or very low (<10 CFU/g). However, some pathogens appeared in higher numbers during storage, though they remained within the standard limits set by the Thai Agricultural Commodity and Food Standard [21], confirming that the samples were safe and not spoiled by pathogens. In AP, a continuous increase in *S. aureus* was observed, reaching 69 CFU/g by Day 12, likely due to the aerobic environment that favors its growth. In contrast, all MAPs effectively inhibited *S. aureus* growth, with levels being undetectable, suggesting that CO_2_ suppresses *S. aureus* by lowering pH and reducing O_2_ availability [44]. Interestingly, *C. jejuni*, which was not detected at Day 0 in AP samples, was found only in M30 and M40 samples. *C. jejuni*, known as anaerobic and microaerophilic pathogens, can adapt to CO_2_-rich environments, which may explain its survival [45]. Our results remind us that while MAP conditions, particularly those consisting of high CO_2_ levels, can extend meat shelf life by suppressing overall microorganisms, they may promote the growth of resistant species like *C. jejuni*, which could impact the safety of the products.

### 3.3. Changes in Quality Attributes of Goat Meat During Storage

The crucial quality attributes of goat meat, including pH, color, TVB-N, TBARSs, and shear force, were monitored throughout storage under different packaging conditions (Figure 5B–F). The pH of goat meat at Day 0 was 5.55 (Figure 5B). It increased slightly by Day 6 with no significant difference among samples (*p* > 0.05), but rose more noticeably by Day 12, with AP reaching the highest pH (5.99) and M40 the lowest (5.66) (*p* < 0.05). This trend could be associated with the metabolic activity of spoilage microorganisms, particularly proteolytic bacteria like *Pseudomonas*, which produce ammonia and amines as byproducts of protein degradation [27]. The lower pH observed in MAP samples suggested that CO_2_-enriched environments may suppress spoilage-related microbial activities, slowing down the accumulation of alkaline metabolites. Additionally, the higher abundance of LAB, acid-producing bacteria, under MAP conditions might also contribute to pH reduction.

Redness is a key indicator of the freshness of red meat and plays a significant role in consumer perception. On Day 12, redness (a*) was significantly reduced in M30 and M40 (*p* < 0.05), while no significant difference was observed between AP and M20 (*p* > 0.05) **(**Figure 5C). The decline in redness in M30 and M40 likely resulted from CO_2_-induced oxidation of oxymyoglobin to metmyoglobin [46], which could plausibly affect consumer perception at the later stages of storage. High CO_2_ concentrations (>30%) are known to negatively impact meat color due to increased oxidative stress, while CO_2_ suppresses microbial growth, excessive levels may affect color stability [47,48].

TVB-N in all samples increased as storage time progressed, with AP exhibiting the most pronounced rise, accumulating higher levels than all MAP samples (*p* < 0.05) (Figure 5C). This increase is attributed to spoilage bacteria breaking down proteins and producing nitrogenous compounds (NH_3_, TMA, DMA), which contribute to undesirable characteristics, particularly off-odor, in stored meat, as reflected by elevated TVB-N levels [23]. This trend corresponded with the fastest increase in TVC in AP (Figure 5A), which exceeded the spoilage thresholds by Day 12, indicating rapid deterioration. After 12 days, bacterial loads in AP had surpassed this threshold, aligning with a sharp rise in TVB-N and signaling advanced spoilage. MAP effectively controlled TVB-N accumulation, suggesting extended shelf life and reduced spoilage under CO_2_-enriched conditions. Among all MAP samples, M40 was the most effective in delaying spoilage, as indicated by the lowest TVB-N value (*p* < 0.05), suggesting high CO_2_ concentrations suppressed spoilage bacteria, thereby limiting the formation of volatile nitrogenous compounds. CO_2_ is known to inhibit key pathogens like *Pseudomonas* and *B. thermosphacta*, reduce enzyme activity, and slow metabolism [10]. Additionally, CO_2_ promotes LAB growth, which outcompete spoilage microbes and produce organic acids that further inhibit proteolysis. However, while CO_2_ initially inhibits bacterial growth, CO_2_-resistant bacteria like Enterobacteriaceae and *Lactobacillus* can adapt over time, accelerating protein degradation [49]. Both endogenous proteases and microbial enzymes contribute to nitrogenous spoilage [50]. Therefore, although microbial activity is slowed at 4 °C in MAP samples, it still occurs, leading to pH fluctuations that enhance proteolytic processes. According to Xia et al. [51], the acceptable TVB-N limit for fresh products is 30 mg/100 g. In this study, TVB-N levels remained within that standard across all samples after 12 days of storage, suggesting that undesirable spoilage characteristics may not have developed significantly during this period.

The TBARS value represents the secondary products of lipid oxidation, which are responsible for the undesirable odor/flavor associated with lipid deterioration, with values between 2.00 and 2.25 mg MDA/kg recognized as the limit indicating no rancidity in meat and meat products [52]. As shown in Figure 5E, the TBARS value remained stable in all MAP samples until Day 6, whereas a gradual increase was observed in AP. However, by Day 12, TBARS levels had clearly increased in all samples, with higher amounts observed in AP and M20 (*p* < 0.05). This suggested that lipid oxidation occurred during storage, particularly after 6 days, and that M30 and M40 were effective in suppressing this reaction. The delayed onset of lipid oxidation may be attributed to the naturally low fat content of goat meat, which initially limits oxidative reactions [53,54]. In addition, CO_2_ concentrations above 30% appeared to suppress lipid oxidation by reducing O_2_ availability and inhibiting oxidative enzymes [55].

Shear force remained unchanged in AP throughout the 12-day storage period Figure 5F, which may be attributed to a balance between oxidative-induced toughness and proteolytic softening. The certain O_2_ of atmosphere condition likely promoted oxidative cross-linking of myofibrillar proteins, helping to maintain firmness despite proteolytic degradation during storage. This corresponded to the statement by Wang et al. [56] that oxidation typically increases meat toughness under air packaging conditions. In contrast, shear force of all MAP samples gradually decreased (*p* < 0.05), suggesting a reduction in firmness over time. The results indicated that CO_2_ retards protein oxidation, thereby allowing proteolytic enzyme activity to predominantly improve tenderness. This suggested that, while CO_2_ slows microbial spoilage, it does not inhibit calpains and cathepsins, which are the main proteolytic enzymes responsible for proteolysis during storage [57]. High CO_2_ reduces O_2_ availability, minimizing oxidative cross-linking of myofibrillar proteins, preventing toughness [58]. It also promotes LAB growth, which lowers pH and enhances proteolytic activity, further tenderizing the meat [57]. Altogether, MAP, particularly with >30% CO_2_, preserved goat meat quality by suppressing spoilage indicators such as TVB-N and TBARSs. Additionally, CO_2_’s ability to modulate protein oxidation, pH levels, and microbial activity contributes to reduced water loss, helping to maintain the meat’s softness and overall quality during storage [59]. However, these conditions (30–40% CO_2_) may have negatively impacted the color stability of high O_2_ MAP, as they accelerated the reduction in redness. The regulation of microbial populations and meat characteristics by CO_2_ levels during storage was observed. Understanding the correlation between them could help optimize CO_2_ levels and improve our understanding of how CO_2_ regulates these changes, both in terms of prolonging shelf life and maintaining desirable characteristics.

### 3.4. Correlation Analysis Between Microbial Communities and Quality Attributes

**Figure 6:**
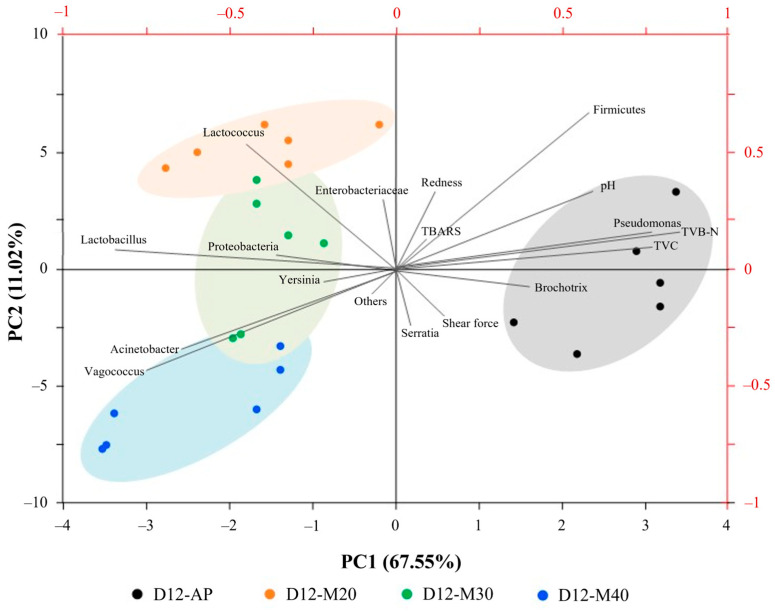
Biplot of a PCA performed on bacterial phyla and genera (relative abundance >5%) and quality attributes of goat meat stored under different MAP conditions at 4 °C on day 12th of storage.

A PCA biplot was used to assess the relationship between the most differentiating microbial abundances at both the phylum and genus levels and the quality attributes of the samples on Day 12 (Figure 6). PC1 (67.55%) clearly separated AP (on the right side) from MAP samples (on the left side), indicating that specific CO_2_ levels (20–40%) in high-O_2_ MAP influenced distinct microbial and quality profiles of goat meat during storage. PC2 (11.02%) further discriminated among the MAP treatments, suggesting that each CO_2_ level shaped unique microbial communities and corresponding quality changes. On the right side of the biplot, a strong positive correlation was observed between TVB-N, TVC, *Pseudomonas*, and *Brochothrix*, which were strongly associated with AP. This suggested that these spoilage-associated bacteria were the primary contributors to protein degradation and nitrogenous compound accumulation in AP, leading to faster deterioration of meat quality during storage. The dominance of *Pseudomonas* in AP aligned with previous findings highlighting its role in aerobic spoilage, particularly through proteolysis and the production of volatile compounds [60]. *Lactobacillus* was the most distinct genus separating MAP samples from AP, appearing at the far left of the axis; however, each MAP sample also exhibited its own dominant genera. *Lactococcus* was positively correlated with M20, suggesting that lower CO_2_ levels may favor the dominance of LAB, which can enhance microbial stability and potentially improve quality by inhibiting spoilage organisms [40]. *Proteobacteria* and *Yersinia* showed a strong correlation with M30, likely reflecting their ability to adapt to CO_2_-enriched environments. *Proteobacteria*, including *Pseudomonas*, are facultative anaerobes capable of thriving under high CO_2_ conditions due to their flexible metabolic pathways [12]. Similarly, *Yersinia*, particularly *Y. enterocolitica*, is known to tolerate elevated CO_2_ levels, allowing it to persist when other microbes are suppressed [61]. These bacteria likely gain a competitive advantage in MAP by exploiting available nutrients and adapting to the CO_2_-rich atmosphere. Moreover, microbial interactions between *Proteobacteria* and *Yersinia* may further support their co-occurrence in this packaging environment. The high CO_2_ concentration, through its pH-lowering and O_2_-reducing effects, likely promotes the dominance of these taxa in 30% CO_2_ MAP. In contrast, the clustering of *Acinetobacter* and *Vagococcus,* which were strongly correlated with M40, suggested the accumulation of distinct microbial genera in the meat after the CO_2_ concentration was increased to 40%. This can be similarly described as their adaptation to CO_2_-enriched, low-O_2_ environments. *Acinetobacter*, a facultative anaerobe, thrives in high CO_2_ concentrations due to its metabolic flexibility [62]. Similarly, *Vagococcus*, a lactic acid bacterium, can tolerate low O_2_ and CO_2_, benefiting from the pH-lowering effects of CO_2_ [35]. Interestingly, the biplot suggested that MAP had a greater influence on microbial composition than on quality attributes, implying that factors beyond microbial presence such as enzymatic activity, oxidative changes, and metabolic byproducts, also play a role in determining meat quality. This study revealed the complex interactions between microbial succession, gas composition, and biochemical processes in MAP-stored goat meat, particularly under elevated CO_2_ levels, emphasizing the need for further in-depth studies into the specific mechanisms driving these changes.

## 4. Conclusions

CO_2_ levels in Hi-O_2_ MAP significantly influenced microbial communities and quality attributes of chilled goat meat over 12 days of storage. Microbial richness and diversity declined across all treatments, with the most rapid deterioration in AP. AP was associated with the dominance of *Pseudomonas* and *Brochothrix,* contributing to higher TVB-N and TBARS levels, indicating faster spoilage. In contrast, MAP, particularly with 40% CO_2_ (M40), suppressed these spoilage bacteria and instead promoted LAB such as *Lactococcus* and *Vagococcus*, enhancing microbial stability. MAP also helped preserve meat texture by limiting oxidative protein cross-linking; however, elevated CO_2_ levels negatively affected redness and overall color stability. Among treatments, M40 provided the most extended microbial stability, though its effect on specific quality traits warrants further study. Future research should assess consumer acceptance and explore microbial metabolic byproducts to better understand CO_2_’s role in shelf life extension and quality maintenance. Storage beyond 12 days should be investigated to determine the actual maximum shelf life. Moreover, monitoring headspace gas dynamics in the packaging should be considered to understand the role of gas ratio changes in relation to microbial and quality changes during storage.

## Figures and Tables

**Table 1:** Richness diversity estimators of bacterial communities in goat meat stored under different MAP conditions at 4 °C for 12 days.

Sample		Read no.	OTUs *	Chao1 **	ACE **	Simpson **	Shannon **	Coverage ***
Day 0		57,340	492	922	980	0.96	6.02	0.9997
Day 6	AP	57,002	489	801	1002	0.96	5.28	0.9997
	M20	55,413	401	800	903	0.93	5.02	0.9998
	M30	54,678	395	789	888	0.95	5.21	0.9997
	M40	55,293	420	822	965	0.95	5.39	0.9998
Day 12	AP	55,465	366	799	993	0.95	5.20	0.9998
	M20	53,890	397	503	785	0.93	4.99	0.9998
	M30	49,299	380	565	703	0.92	4.89	0.9999
	M40	50,400	397	745	902	0.95	5.55	0.9998

* OTUs: operational taxonomic units; ** Chao1, ACE (abundance-based coverage estimator); Simpson and Shannon are alpha diversity indices used to assess species richness and diversity within a sample. *** Coverage indicates how completely microbial diversity is captured by sequencing.

**Table 2:** Pathogenic microorganism counts in goat meat stored under different MAP conditions at 4 °C for 12 days.

Sample		*E. coli*	*S. aureus*	*Salmonella* spp.	*C. perfringens*	*C. jejuni*
Day 0		<10 CFU/g	ND *	ND	<10 CFU/g	ND
Day 6	AP	<10 CFU/g	30 CFU/g	ND	<10 CFU/g	ND
	M20	<10 CFU/g	ND	ND	<10 CFU/g	ND
	M30	<10 CFU/g	ND	ND	<10 CFU/g	ND
	M40	<10 CFU/g	ND	ND	<10 CFU/g	<10 CFU/g
Day 12	AP	53 CFU/g	69 CFU/g	ND	<10 CFU/g	ND
	M20	<10 CFU/g	ND	ND	<10 CFU/g	ND
	M30	<10 CFU/g	ND	ND	<10 CFU/g	<10 CFU/g
	M40	<10 CFU/g	ND	ND	<10 CFU/g	22 CFU/g

* ND = not detected.

## Data Availability

The original contributions presented in the study are included in the article/Supplementary Material, further inquiries can be directed to the corresponding author.

## Supplementary Materials

The following supporting information can be downloaded at: https://www.mdpi.com/article/10.3390/foods14111837/s1. Table S1. Annotated OTUs of bacterial communities in goat meat stored under different MAP conditions at 4 °C for 12 days.
