# Supplementary material for: The Role of CO2 Levels in High-Oxygen Modified Atmosphere Packaging on Microbial Communities of Chilled Goat Meat During Storage and Their Relationship with Quality Attributes
Source: Foods. 2025 May 22;14(11):1837. doi: 10.3390/foods14111837 (PMC12154290; doi:10.3390/foods14111837)
Supplement: Supplementary file 1 [file foods-14-01837-s001.zip › foods-3656854-supplementary.pdf]

Table S1 Annotated OTUs of bacterial communities in goat meat stored under different MAP conditions at 4°C for 12 days.

**Sample**		**Phyla**	**Classes**	**Orders**	**Families**	**Genera**	**Species**
Day-0		40	92	235	288	560	690
Day-6	AP	44	86	244	314	555	702
	M20	39	89	201	299	602	819
	M30	43	79	202	278	508	724
	M40	45	90	243	306	622	844
Day-12	AP	39	79	264	290	543	623
	M20	37	84	236	267	499	606
	M30	42	88	205	271	508	702
	M40	40	94	234	295	601	771
**Total OTUs**		**47**	**101**	**299**	**472**	**889**	**1253**
